# Coupling Relationship Analysis between Quality Infrastructure and Ecological Environment Quality for Policy Implications

**DOI:** 10.3390/ijerph17207611

**Published:** 2020-10-19

**Authors:** Jing Shen, Yang Zhang, Benhai Guo, Suli Zheng

**Affiliations:** 1 College of Economics & Management, China Jiliang University, Hangzhou 310018, China; shenjing@cjlu.edu.cn (J.S.); guobenhai@163.com (B.G.); sl_zheng@126.com (S.Z.); 2 Key Laboratory of Quality Infrastructure Efficacy Research, AQSIQ, Beijing 100028, China

**Keywords:** coupling relationship, quality infrastructure, ecological environment quality, policy implications

## Abstract

Long-term improvement of ecological environment quality (EEQ) is a hotspot and urgent topic in the context of high-quality and sustainable development. It is urgent to look for methods that could support EEQ improvement in a high-quality and sustainable way. Owing to its natural supporting and guaranteeing functions for enhancing quality, quality infrastructure (QI) is a fundamental and critical element in promoting EEQ, but a neglected one. In this paper, we analyzed the coupling structure between QI and EEQ and applied an improved coupling model to recognize contributed and weakened indicators that affected the coupling relationship. We also examined this coupling relationship in the Yangtze River Delta (YRD) from 2012 to 2017, which proved the current situation where QI construction cannot satisfy the practical needs of EEQ improvement. Results showed that the important supporting role of QI in EEQ improvement should be valued for long-term sustainable development. Meanwhile, equilibrium and consistency of indicators in the QI and EEQ systems determined the coupling state. EEQ improvement countermeasures were also provided according to the coupling relationship analysis results. This study provided a scientific basis and guidance for EEQ improvement and sustainable development.

## 1. Introduction

As a thorough implement of ecological civilization in China, pollution prevention and control has made key progress, and ecological environment quality (EEQ), which reflects the suitability of an ecological environment for human survival and sustainable development of a social economy in a specific time and space range, has improved notably. However, the pressure of ecological environment protection in China has not been relieved fundamentally, and the severe situation of environmental pollution and ecological protection has not been changed fundamentally [1]. There are still some weaknesses in laws, regulations, and practices in the process of ecological environment management, impeding high-quality and sustainable development. It is, therefore, urgent to look for methods that could support EEQ improvement in a high-quality and sustainable way.

Owing to its natural supporting and guaranteeing functions for enhancing quality, quality infrastructure (QI) is a basic and critical element in promoting EEQ through effective enforcement of technical regulations and improvement of environmental protection hardware facilities (inspection and testing equipment, laboratory, etc.) and software facilities (legal framework, management system, etc.) [2]. It is a complete quality system that relies on metrology, standardization, and accreditation (which comprises testing, inspection, certification, and accreditation). It comprises organizations (public and private), policies, relevant legal and regulatory frameworks, and practices [3]. In the strict supervision field of ecological environment protection, the bottom goal of ecological environment governance can be achieved through legal measures, mandatory standards, compulsory certification, legal inspection, etc. In the monitoring, prevention, control, management, research, and other technical works of environmental protection, all the instruments and equipment and analytical methods, together with the determination of standard substances and their quantities, must be verified through measurement. Environmental standards are an embodiment of environmental protection planning and the basis of environmental administration. Inspection, testing, certification, and accreditation activities can prevent products that threaten environmental safety from entering the market and maintain the ecological environment in a sustainable development state. Consequently, QI can be an effective tool for improving existing weaknesses in law, regulations, and practices in the EEQ improvement process. It is important to analyze the interrelationship and interdependence between QI and EEQ. The first step is to evaluate the coupling relationship between QI and EEQ.

“Coupling” is a concept that originates from physics, describing the interaction and interdependence between two systems or among more than two systems [4]. Most of the previous research on coupling analysis focused on the interrelationship among miscellaneous attributes in a system or between systems. However, the undeniable fact is that the effectiveness of system attributes on a system object can respect a coupling relationship more authentically with an improved coupling model, which accords with the relationship between QI and EEQ.

Many multilateral relationship analyses that take EEQ into account have been conducted with a society, economy, resources, or energy system [5,6,7,8]. Just a few studies have tried to reveal the essential and foundational role of the three elements of QI (standards, metrology, and accreditation) with respect to EEQ [9,10]. However, most have revealed the interactions between a single element of QI and EEQ. For instance, Goulden et al. [11] stated that standards, as a tool for regulating environmental issues, play a key role in defining acceptable levels of safety and environmental protection. Kang et al. [12] found that a typical metrological tool can be applied to cope with environmental issues and meet environmental regulations within the electronics industry. Orcos et al. [13] expressed that the number of ISO 14001 certifications is higher in countries where stakeholders exert stronger pressure on firms to control their environmental quality. Overall, scholars have focused more on the relationship between the three elements of QI and EEQ [14,15,16,17,18]. However, just a few of existing studies have focused on the relationship between QI and EEQ with a more systematic and comprehensive perspective that takes all the three elements into account within the QI system.

Basing on previous studies, this study has three main purposes: (i) identifying the relationship structure between QI and EEQ, (ii) determining appropriate and applicable methods to quantify the coupling relationship between QI and EEQ, and (iii) providing policy implications to support EEQ improvement from the viewpoint of QI in a sustainable and high-quality way. For these purposes, three steps that include coupling structure analysis, index system construction, and coupling model application were conducted. A case study of the Yangtze River Delta (YRD) was conducted based on historical data. The findings of this study may not only help the mutual promotion between a QI system and a EEQ system but also provide a scientific basis for further sustainable and high-quality development planning. To our knowledge, this is the first study to analyze the interrelationship between QI and EEQ.

## 2. Materials and Methods

The purpose of this paper was to analyze the coupling relationship between QI and EEQ to present policy implications for decision makers and eco-environment managers. For this purpose, an analysis of the coupling relationship between QI and EEQ was performed, which included three steps: (1) coupling structure analysis for QI and EEQ in terms of their nature interrelationship, (2) index system construction for QI and EEQ according to their coupling structure, and (3) coupling model application to achieve a coupling degree between QI and EEQ and recognition of contributed and weakened indicators that affected the coupling relationship. 

### 2.1. Coupling Structure Analysis

**Figure 1:**
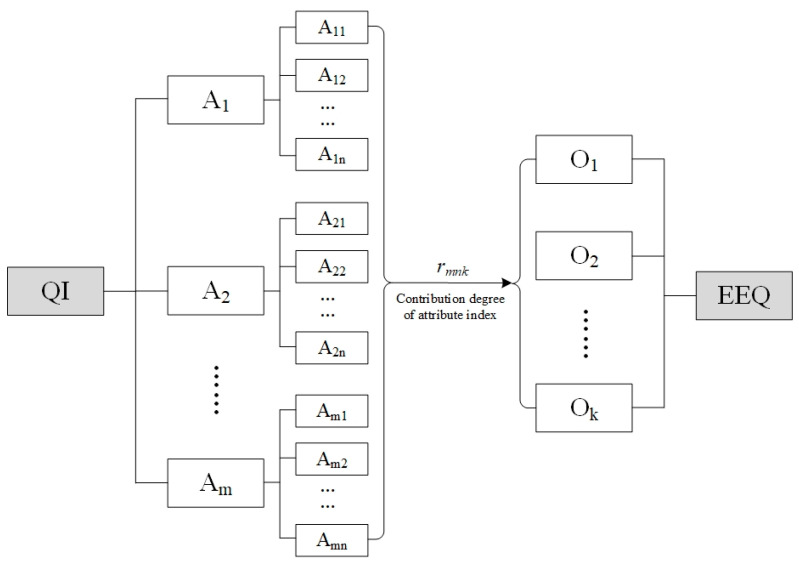
Coupling structure of a ““attribute–object” pattern between QI and EEQ.

A QI system, as public goods, has targets to improve quality development in all aspects of human society. EEQ improvement, as one of the targets of QI construction, requires support from regulation and constraint of environmental standards, technical support from metrology on environmental monitoring, and technological capability promotion from accreditation. The relationship between QI and EEQ presents a pattern of “attribute–object.” Therefore, a traditional coupling model, which considers only the interrelationship between two or more horizontal systems, is not appropriate for analyzing the relationship between QI and EEQ. Instead, an improved coupling model built by Wang et al. [19] that focuses on the analysis of the coupling relationship between system attributes and a system object is in accord with the relationship between QI and EEQ. The coupling structure of these two systems is shown in Figure 1, *A_1_*, *A_2_*,..., *A_m_* are *m* sub-attribute systems of QI; *A_m1_*, *A_m2_*,..., *A_mn_* are *n* attributes of a subsystem; and *S_m_*, *O_1_*, *O_2_*,..., *O_k_* are *k* sub-objects of EEQ.

### 2.2. Index System Construction

The assessment index system should be constructed synthetically and veritably to represent all the aspects of a QI system and EEQ system and satisfy the “attribute–object” coupling structure. In this paper, the construction principles of goal orientation, systematicness, objectivity, rationality, feasibility, and representation were complied with. The index system construction included four steps. First, an in-depth literature review guided by the QI and EEQ research above was applied to discern relevant data sets for consideration [20,21,22,23]. Data were collected from a yearbook of general administration of quality supervision, inspection, and quarantine of the People’s Republic of China; a statistical communique on national economic and social development; a Chinese urban construction statistical yearbook, and a statistical yearbook on environment. A total of 18 relevant indices for the EEQ system and 54 relevant indices for the QI system were first selected based on literature review and data availability. Second, Pearson correlation analysis and rotated principal component analysis were performed to reduce redundancy [24,25]. One index was discarded for its highly correlated pair of indices (|r| > 0.9), and high factor loading indices (|r| > 0.75) were kept. The number of EEQ and QI indices was reduced to 13 and 37, respectively. Then, expert investigation method was used to determine eventual indicators that summarize and reflect the real situation of QI and EEQ. Ten experts were selected from the quality administration and measurement departments, standard institutes, and related scholars [26]. The 10 experts judged the indices in five aspects: scale appropriateness, suitability, measurability, scientific validation, and discriminating ability. Each indicator was assigned a score by the 10 experts from 1 (very unfit) to 5 (very fit) regarding its relevance to each criterion. Then, the average score was calculated for each indicator, during which equal weights were given to all the experts and selection criteria. Equal weights were applied because the relevance scores had no significant differences between the criteria (*p* = 0.12) and the experts (*p* = 0.07). The indicators that received very low values from one or more experts were discarded. When one or more experts pointed out severe shortcomings associated with certain indicators, the indices were also discarded. Similar (identical nature but different expression) indices were also discarded after panel discussion. The top-scored indices were kept per criterion. Finally, 23 indices were taken into account to evaluate the QI system as an attribute system (shown in Table 1), including the four subsystems of infrastructure status, metrology, standardization, and accreditation. Seven sub-objects were included to represent the EEQ system as an objective system (shown in Table 2). These selected indicators had the characteristics of scientific nature, widespread acceptance, and available data collection.

### 2.3. Coupling Model between QI and EEQ

#### 2.3.1. Contribution Degree of Attribute Index

The coupling relationship between QI and EEQ may be indicated by the contribution degree of the QI attribute indices (shown in Table 1) to the EEQ sub-objects (shown in Table 2). If data from the QI attribute index *A_mn_* in time *t* is *x(t)* (*t* = 1,2,...,*h*), then the set of *X_mn_* = (*x(1)*, *x(2)*,..., *x(h)*) is a behavior sequence of the QI attribute index *A_mn_*. Meanwhile, *Y_k_* = (*y(1)*, *y(2)*,...,*y(h)*) is a behavior sequence of the EEQ sub-object *O_k_*. Considering that the raw data from multiple QI attributes and EEQ objects have their own evaluation unit, the data from the QI attribute indices and EEQ sub-objects were standardized by the mean value nondimension method shown in Equations (1) and (2), respectively.

(1) $$X^{\prime }=\frac{x(t)}{\bar{x(t)}}$$

(2) $$Y^{\prime }=\frac{y(t)}{\bar{y(t)}}$$

Then, the relational coefficient of each QI attribute index to the EEQ sub-subject can be calculated by Equation (3) as follows:

(3) $$\zeta {\left(t\right)}^{*}=\frac{minmin\left|x^{\prime }(t)-y^{\prime }(t)\right|+\rho maxmax\left|x^{\prime }(t)-y^{\prime }(t)\right|}{\left|x^{\prime }(t)-y^{\prime }(t)\right|+\rho maxmax\left|x^{\prime }(t)-y^{\prime }(t)\right|}$$

where *ρ* is the distinguished coefficient, *ρ* ∈ [0,1], and is set as 0.5.

The relational degree between the QI attribute index and the EEQ sub-object can be defined as:

(4) $$r(x^{\prime },\;y^{\prime })=\frac{1}{h}\sum_{t=1}^{h}\zeta {\left(t\right)}^{}$$

Thus, the contribution of the QI attribute index in the EEQ sub-object can be expressed by the relational degree between the QI attribute index and the EEQ sub-object as:

*r_mnk_* = *r*(*A_mn_*,*O_k_*) (5)

It can be seen from the equations that a bigger relational degree between the QI attribute index and the EEQ sub-object means more contribution of the QI attribute index to the EEQ sub-object. Conversely, a smaller relational degree indicates less contribution of the QI attribute index to the EEQ sub-object.

#### 2.3.2. Contribution Degree of the Sub-Attribute System

In the QI sub-attribute system *A_m_*, there are *n* QI attribute indices. As the QI attribute index contributes to the EEQ sub-object with *r_mnk_*, the contribution degree of the QI sub-attribute system to the EEQ sub-object can be calculated by the contribution degree of the QI attribute indices with the geometric mean as follows:

(6) $$R_{mk}=\sqrt[{n}]{r_{m_{1}k}\cdot r_{m_{2}k}\cdot \cdot \cdot \cdot \cdot r_{m_{n}k}}={\left(\prod_{n=1}^{n}r_{m_{n}k}\right)}^{\frac{1}{m_{n}}}$$

#### 2.3.3. Improved Coupling Model

In physics, coupling refers to a phenomenon in which two or more systems or movement forms influence another through various interactions. A contribution matrix of QI to EEQ can be obtained from Equation (7) as follows:

(7) $$\left[\begin{array}{cccc} R_{11} & R_{12} & \cdots \cdots & R_{1k}\\ R_{21} & R_{22} & \cdots \cdots & R_{2k}\\ \vdots & \vdots & \vdots & \vdots \\ R_{m1} & R_{m2} & \cdots \cdots & R_{mk} \end{array}\right]$$

As the contributions of each QI sub-attribute to multiple EEQ sub-objects are different, the coupling degrees between QI and EEQ will be better accompanied by fewer differences among the contribution degrees of four QI sub-attributes to the seven EEQ sub-objects. On the contrary, the coupling degree between QI and EEQ will be worse accompanied by bigger differences among the contribution degrees. Thus, the coupling degree between QI and EEQ can be expressed by the variance of the contribution degree (*C*) of the QI sub-attribute to the EEQ sub-object by Equation (8). It means that, with a smaller *C*, the coupling level between QI and EEQ will be better. Meanwhile, the coupling level will be worse with a bigger *C*.

(8) $$C=\sqrt[{}]{\frac{1}{k}\sum_{i=1}^{k}{\left(\lambda_{i}-\bar{\lambda_{}}\right)}^{2}}$$

(9) $$\lambda_{i}=\sqrt[{}]{R_{1i}^{2}+R_{2i}^{2}+\cdot \cdot \cdot \cdot \cdot +R_{ni}^{2}}={\left[\sum_{j=1}^{m}{\left(\prod_{n=1}^{n}r_{j_{n}i}\right)}^{\frac{2}{n}}\right]}^{\frac{1}{2}}$$

where *λ_i_* is the module of a vector (*R_1i_*, *R_2i_*, ..., *R_nk_*)*^T^*, which represents the contribution degree of each QI sub-attribute system to the *i_th_* EEQ sub-object.

In *λ_1_*, *λ_2_*,... *λ_i_*, the *i_th_* EEQ sub-object would be the weakened EEQ indicator for the QI–EEQ coupling relationship with the biggest value of |*λ_i_* − *λ*|, which means that this EEQ sub-object has the worst coupling degree with the QI attribute system. Conversely, the *i_th_* EEQ sub-object would be the contributed object indicator with the smallest value of |*λ_i_* − *λ*|, which means that this EEQ sub-object has the best coupling degree with the QI attribute system.

To find the weakened QI sub-attribute system and attribute index, there are two situations: (1) if *λ_i_* − *λ <* 0, for the weakened EEQ indicator, the weakened QI sub-attribute system can be found with the smallest value of the contribution degree of the QI sub-attribute system among *R_1i_*, *R_2i_*, ...*R_mi_*, and the weakened QI attribute index can be sought out with the smallest value of the contribution degree of the QI attribute index among *r_m1i_*, *r_m2i_*, ...*r_mni_* in the weakened QI sub-attribute system; (2) if *λ_i_* − *λ >* 0, for the weakened EEQ indicator, the weakened QI sub-attribute system can be found with the biggest value of the contribution degree of the QI sub-attribute system among *R_1i_*, *R_2i_*, ...*R_mi_*, and the weakened QI attribute index can be sought out with the biggest value of the contribution degree of the QI attribute index among *r_m1i_*, *r_m2i_*, ...*r_mni_* in the weakened QI sub-attribute system.

### 2.4. Case Study

**Figure 2:**
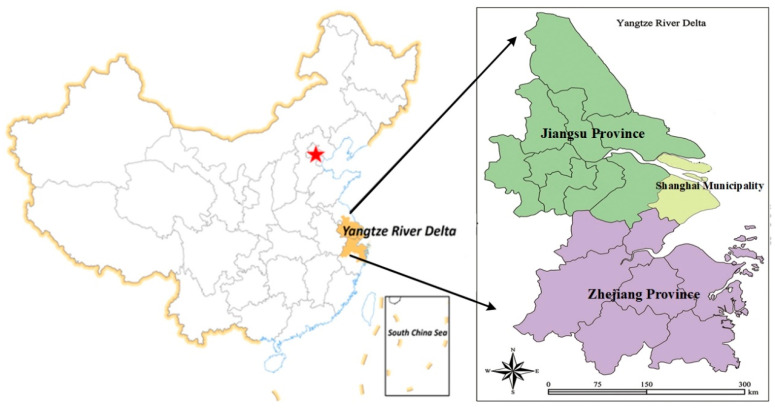
Location and administrative divisions in the YRD region.

The YRD, including Jiangsu province, Zhejiang province, and Shanghai municipality, is one of the most dynamic, open, and innovative regions in China and one of the six world-class city groups (shown in Figure 2). The YRD is characterized by a marine monsoon subtropical climate. The YRD covers an area of 217,700 km^2^ and is located at 29°54.3′–32°6.15′ N, 120°13.6′–121°0.143′ E. Until 2018, the residential population of the YRD was 162 million, which accounted for approximately 7.8% of all Chinese people. This region occupies 2.1% of the Chinese territory, while generating a billion Chinese yuan (CNY) in 2018, 20.16% of the total national gross domestic product (GDP).

The general situation of QI in the YRD takes a leading position in China. More specifically, the standardized resources and standardized service level in all cities in the YRD were all top 5 in rank by certification bodies and certificates among 34 provincial administrative regions. The total numbers of certification bodies and certificates in the YRD were 701,714 and 208,100, respectively, with percentages of 30.06% and 31.61% countrywide in 2019. The percentage of the qualified rate of environmental measuring instruments with compulsory inspection in all cities in the YRD was 13.16%, at the forefront in China.

## 3. Results

According to the coupling relationship analysis method shown in Section 2, data from the attribute index in the QI system and the sub-object in the EEQ system from 2012 to 2017 in the YRD region were collected. The coupling degrees (*C*) for Zhejiang province, Shanghai municipality, Jiangsu province were 0.1330, 0.1298, 0.150, respectively, presenting a rank of Shanghai municipality < Zhejiang province < Jiangsu province. From the results, it can be seen that, in the YRD region, the coupling relationship between QI and EEQ was best accompanied in Shanghai municipality with the smallest differences among the contribution degrees of four QI sub-attribute systems to the seven EEQ sub-objects. The second was Zhejiang province, and the last was Jiangsu province, with the biggest differences among the contribution degrees of four QI sub-attribute systems to the seven EEQ sub-objects’ variance of contribution degrees. In the following section, a weakened EEQ sub-object, weakened QI sub-attribute system, and weakened QI attribute index were recognized by the methodologies shown in Section 2.3.3 and described which could provide scientific support for further countermeasures to improve EEQ through consolidation and enhancement of QI.

### 3.1. Weakened EEQ Sub-Object Recognition

Table 3 presents the rank of the EEQ sub-objects in the YRD region. The results indicate that the weakened EEQ object in the three provincial administrative units in the YRD region were different and illustrated the diverse coupling relationship between QI and EEQ. In Zhejiang province, Shanghai municipality, and Jiangsu province, the weakened EEQ object with the biggest value of $\left|\lambda_{i}-\bar{\lambda_{}}\right|$, which was most disadvantageous for the coupling relationship between QI and EEQ, were O_4_ (acid rain frequency), O_2_ (qualified rate of surface water quality), and O_4_ respectively. Meanwhile, the most attributed EEQ objects for the coupling relationship in the three administrative units were O_5_ (ecological index), O_3_ (fine rate of atmospheric environment quality), and O_5_ (ecological index).

The results also indicate that the QI system had not shown an obvious supporting role in EEQ improvement, and existing QI had a better coupling relationship with the EEQ sub-objects that had little changes in historical data in the YRD region. According to the coupling relationship analysis results, the EEQ sub-objects with an obvious improvement trend in historical data were the major weakened factors for the coupling relationship between QI and EEQ. Instead, the sub-objects with little change in 6 years had more contribution for the coupling relationship between QI and EEQ. For instance, in Zhejiang province, the seven weakened EEQ sub-objects listed in descending order were acid rain frequency, qualified rate of drinking water quality, urban sewage treated ratio, fine rate of atmospheric environment quality, qualified rate of surface water quality, percentage of harmless disposal of domestic garbage, and ecological index. However, the first six sub-objects presented an improvement trend in EEQ according to the historical data, and the seventh sub-object had little changes. The same situations also applied to Shanghai municipality and Jiangsu province.

### 3.2. Weakened QI Sub-Attribute System Identification

Table 4 presents the contribution degree matrix of the QI sub-attribute system to the EEQ sub-objects in the YRD region and the recognition basis for the weakened QI sub-attribute system. Based on the identification principles shown in Section 2.3.3, the weakened EEQ sub-object in Zhejiang province was O_4_ (acid rain frequency), and *λ_4_ − λ of* O_4_ was less than 0, which is shown in Table 3. Therefore, the QI sub-attribute system A_1_ (infrastructure status), with the smallest value of 0.3062 among the four values in the fifth column of the first matrix in Table 4, was the weakened QI sub-attribute system to the EEQ sub-object. In the same way, the weakened QI sub-attribute systems of Shanghai municipality and Jiangsu province can be found in M (metrology) and A (accreditation), respectively.

### 3.3. Weakened QI Attribute Index Recognition

**Figure 3:**
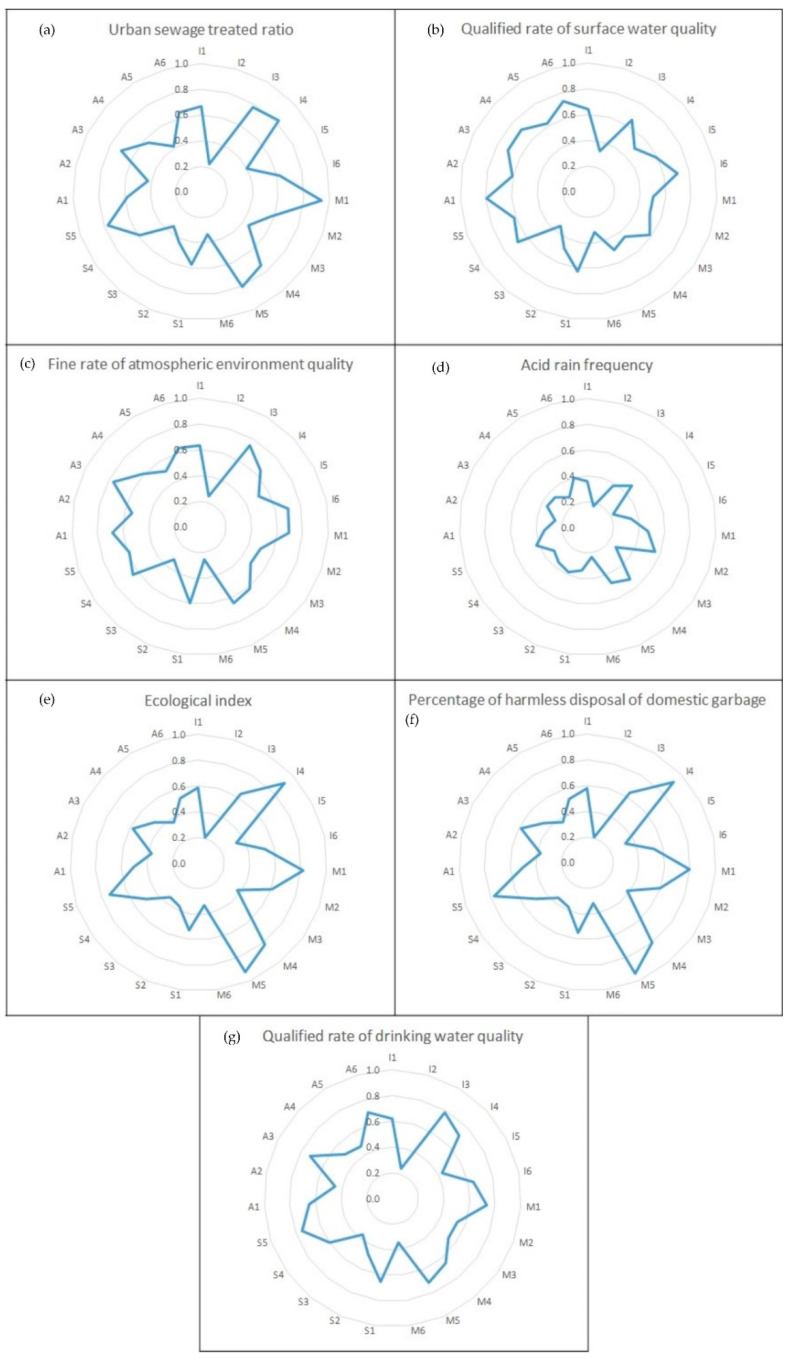
Contribution degree of QI attribute indices to (**a**) urban sewage treated ratio; (**b**) qualified rate of surface water quality; (**c**) fine rate of atmospheric environment quality; (**d**) acid rain frequency; (**e**) ecological index; (**f**) percentage of harmless disposal of domestic garbage; (**g**) qualified rate of drinking water quality in Zhejiang.

**Figure 4:**
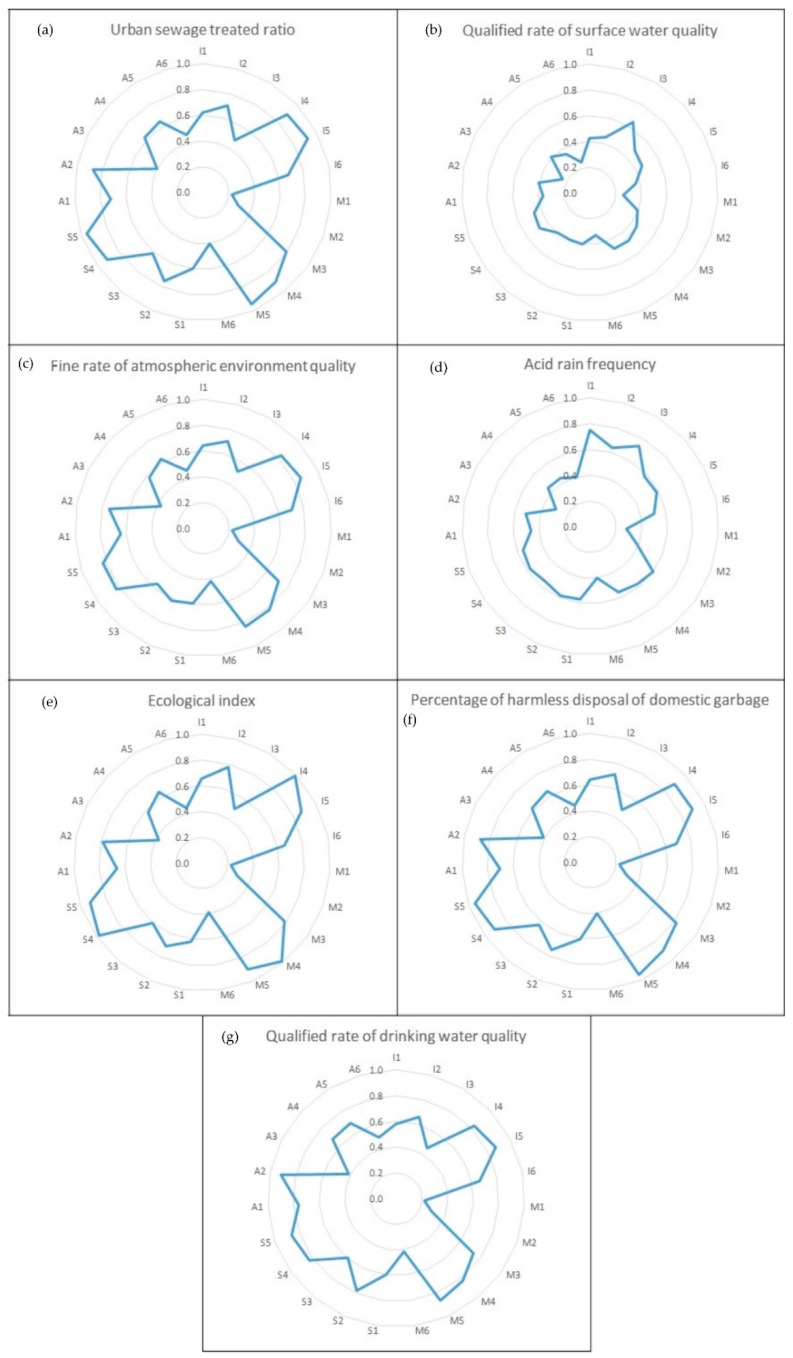
Contribution degree of QI attribute indices to (**a**) urban sewage treated ratio; (**b**) qualified rate of surface water quality; (**c**) fine rate of atmospheric environment quality; (**d**) acid rain frequency; (**e**) ecological index; (**f**) percentage of harmless disposal of domestic garbage; (**g**) qualified rate of drinking water quality in Shanghai.

**Figure 5:**
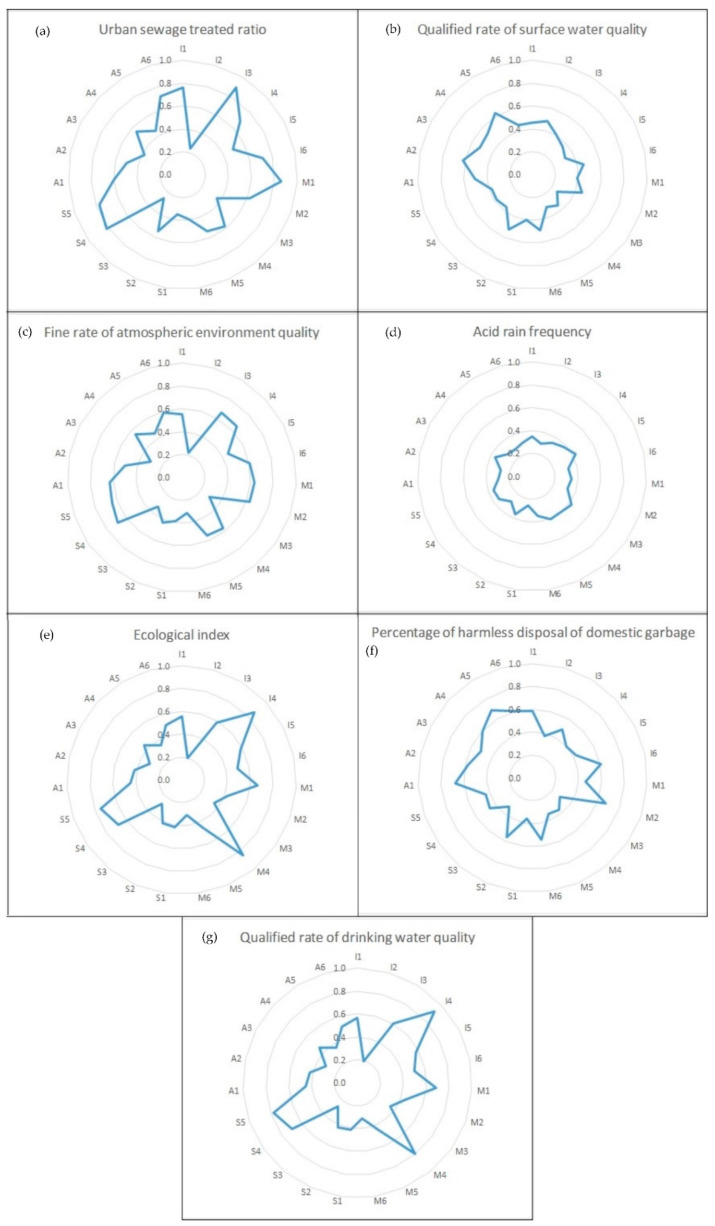
Contribution degree of QI attribute indices to (**a**) urban sewage treated ratio; (**b**) qualified rate of surface water quality; (**c**) fine rate of atmospheric environment quality; (**d**) acid rain frequency; (**e**) ecological index; (**f**) percentage of harmless disposal of domestic garbage; (**g**) qualified rate of drinking water quality in Jiangsu.

The contribution degrees of the QI attribute indices to the EEQ sub-objects in Zhejiang province, Shanghai municipality, and Jiangsu province are shown in Figure 3, Figure 4 and Figure 5, Respectively. Following the above-identified weakened QI sub-attribute system, the weakened QI attribute index can be found through the principles shown in Section 2.3.3. Taking Zhejiang province as an example, the weakened EEQ sub-object in Zhejiang province was O_4_ (acid rain frequency) with *λ_4_ − λ <* 0, and I (infrastructure status) was the weakened QI sub-attribute system. So the weakened QI attribute index I_4_ (green ratio for built-up area) can be sought out with the smallest value of the contribution degree of the QI attribute index in Figure 3d. The weakened QI attribute index of Shanghai municipality and Jiangsu province can be found in M_1_ (numbers of quality supervision department) and A_5_ (numbers of national certification and accreditation laboratories), respectively, in the same way.

### 3.4. Countermeasures

High-quality and sustainable development needs a coordinated development between QI and EEQ. With a pattern of “attribute–object,” the equilibrium and consistency of the indicators between QI and EEQ determine the coupling state of the two systems. The case study in the YRD proved the current situation where QI construction cannot satisfy the practical needs of EEQ improvement. In terms of the low social consciousness [27] and late start of QI concept analysis and research, especially lack of recognition of QI’s strategic and fundamental supporting role, QI construction in China is affected and limited to some extent. Meanwhile, there is a QI gap between Chinese and international advanced levels, with void in some core QI technologies. Moreover, QI involves various departments and a complex management system, which has not established a comprehensive coordination mechanism with ecological environment protection departments. The results of the coupling relationship analysis can help decision makers comprehend principle contradictions in environmental management. Promoting the coupling relationship of QI and EEQ needs to start from the following aspects:

(1) The QI system and policy should be constructed and updated to cope with demand for sustainable development and EEQ improvement. The QI system and policy should both reflect the current national and international environments and possess perspectiveness and predictability. Current regulation, especially regarding environmental protection standards, legal metrology for environmental monitoring instruments, and accreditation, needs to be updated and refined. Meanwhile, the national calibration system in China should be added to regulations as the current calibration system has not been outlined in applicable regulations, which means that there are no regulations to constrain measuring instrument calibration in environmental monitoring.
(2) QI technological foundation should be consolidated to improve the efficiency of ecological environment management. More specifically, we should deeply implement the strategy of innovation-driven development, cultivate the innovation vitality of QI institutions, accelerate the technical support capacity and innovation-leading ability of QI institutions, increase the research and development of QI technology and equipment, and comprehensively improve the service ability of QI institutions for ecological environment management. Moreover, the organic combination of QI and big data could provide powerful technical support for EEQ improvement.
(3) QI as a tool can be used in ecological red-line delineation, baseline defense, and ecological civilization construction. With the support of a certification system, metrology, and testing technique, enterprise behavior can be effectively restrained by energy consumption standards, environmental protection standards, compulsory verification of environmental monitoring measuring instruments, compulsory product certification, and legal inspection. Consequently, QI can be a useful tool to improve the application efficiency of resources and energy and reduce emission of carbon dioxide and pollutants.
(4) The QI chain management of “metrology–standard–accreditation” in the production process should be improved to promote a comprehensive utilization rate of resources and realize eco-friendly production. From the viewpoint of clean and green production, construction and strengthening of (a) a metrological monitoring system; (b) a standard system for energy saving, emission reduction, and circular economy; and (c) an accreditation system for low-carbon production identification and energy conservation products can effectively control pollutant production and discharge in the production process and substantially reduce resource consumption. It is helpful to resolve overcapacity and realize sustainable development.

## 4. Conclusions

Overall, this paper analyzed the coupling structure between QI and EEQ with a pattern of “attribute–object” and built an assessment index system that represents their coupling relationship. On this basis, contributed indicators and weakened indicators that affect this coupling relationship could be recognized by applying an improved coupling model in the empirical study. The results indicate that equilibrium and consistency of indicators between the QI and EEQ systems determined the coupling state. Coupling relationship analysis between QI and EEQ is crucial for sustainable development. It can help decision makers to comprehend principle contradictions in the EEQ enhancement process from viewpoint of the QI system and provide a scientific basis to make countermeasures.

The case study in the YRD proved the current situation where QI construction cannot satisfy the practical needs of EEQ improvement. Owing of the low social consciousness and late start of QI concept analysis and research, especially lack of recognition of QI’s strategic and fundamental supporting role, QI construction in China is affected and limited to some extent. Meanwhile, there is a QI gap between Chinese and international advanced levels with blank in some core QI technologies. Moreover, QI involves various departments and a complex management system, which has not established a comprehensive coordination mechanism with ecological environment protection departments. Therefore, the important supporting role of QI for EEQ improvement should be valued for long-term sustainable development.

This paper focused on coupling relationship analysis and results representation by using an assessment index system and improved coupling model. The constructed coupling model between QI and EEQ can only recognize contributed and weakened indicators for the past and current situations and cannot predict the future coupling status. In addition, this model supposed that the weights of all the QI attribute indicators for each EEQ sub-object were equal, thereby neglecting weight differences. Meanwhile, it lacked in-depth exploration of the coupling mechanism between QI and EEQ. In further studies, this coupling mechanism may be analyzed quantitatively and qualitatively with a method for system dynamics among various systems, which could simulate EEQ improvement path and select the optimal development route. Research scale can also be broadened to cover more cities, provinces, and even countries for a deeper investigation and wider comparison, which could provide straightforward help for decision makers to better solve local practical problems.

## Figures and Tables

**Table 1:** The index system of QI.

Attribute System	Sub-Attribute System	Attribute Index	Data Sources
QI	Infrastructure status (I)	I_1_: Professional technician numbers	(1)
		I_2_: Total value of fixed assets	(1)
		I_3_: Employee numbers at the end of the year	(1)
		I_4_: Green ratio for built-up area	(2)
		I_5_: Pro-environment input intensity	(2)
		I_6_: Working room area	(1)
	Metrology (M)	M_1_: Numbers of quality supervision department	(1)
		M_2_: Numbers of measuring instruments	(3)
		M_3_: Numbers of measurement standards	(3)
		M_4_: Qualified rate of measuring instruments with compulsory inspection	(3)
		M_5_: Qualified rate of measuring instrument performance	(3)
		M_6_: Numbers of measuring instruments with compulsory inspection for environmental monitoring	(3)
	Standard (S)	S_1_: Numbers of international standards	(3)
		S_2_: Contribution index for national standards	(4)
		S_3_: Contribution index for industry standards	(4)
		S_4_: Numbers of technical committee	(3)
		S_5_: Numbers of collection standards	(5)
	Accreditation (A)	A_1_: Numbers of national inspection and detection agency	(3)
		A_2_: Numbers of national quality inspection center	(3)
		A_3_: Numbers of provincial quality inspection center	(3)
		A_4_: Numbers of national calibration and testing bodies	(3)
		A_5_: Numbers of national certification and accreditation laboratories	(6)
		A_6_: Numbers of certification and accreditation bodies	(6)

Note: Detailed information of data sources: (1) scientific statistical yearbook, (2) statistical yearbook, (3) yearbook of general administration of quality supervision, inspection, and quarantine of the People’s Republic of China, (4) national standard information sharing infrastructure, (5) standard library, (6) yearbook of certification and accreditation of China.

**Table 2:** The index system of EEQ.

Objective System	Sub-Object	Data Sources
EEQ	O_1_: Urban sewage treated ratio	(1)
	O_2_: Qualified rate of surface water quality	(2)
	O_3_: Fine rate of atmospheric environment quality	(2)
	O_4_: Acid rain frequency	(2)
	O_5_: Ecological index	(3)
	O_6_: Percentage of harmless disposal of domestic garbage	(1)
	O_7_: Qualified rate of drinking water quality	(2)

Note: Detailed information of data sources: (1) statistical communique on national economic and social development, (2) statistical yearbook on environment, (3) eco-environment quality report of China.

**Table 3:** The ranks of the weakened EEQ sub-objects in the YRD region.

Rank	Zhejiang Province		Shanghai Municipality		Jiangsu Province	
	$\left|\lambda_{i}-\bar{\lambda_{}}\right|$	Sub-Object	$\left|\lambda_{i}-\bar{\lambda_{}}\right|$	Sub-Object	$\left|\lambda_{i}-\bar{\lambda_{}}\right|$	Sub-Object
1	(0.3125)	O_4_	(0.2444)	O_2_	(0.2433)	O_4_
2	0.1014	O_7_	(0.1510)	O_4_	0.1526	O_1_
3	0.0921	O_1_	0.1035	O_6_	(0.0582)	O_2_
4	0.0763	O_3_	0.0982	O_1_	0.0566	O_6_
5	0.0382	O_2_	0.0966	O_5_	0.0519	O_3_
6	0.0028	O_6_	0.0718	O_7_	0.0260	O_7_
7	0.0016	O_5_	0.0252	O_3_	0.0144	O_5_

Note: Detailed information of the sub-object: O_1_: urban sewage treated ratio; O_2_: qualified rate of surface water quality; O_3_: fine rate of atmospheric environment quality; O_4_: acid rain frequency; O_5_: ecological index; O_6_: percentage of harmless disposal of domestic garbage; O_7_: qualified rate of drinking water quality.

**Table 4:** The contribution degree the matrix of the QI sub-attribute system to the EEQ sub-object in the YRD region.

QI Sub-Attribute System	O1	O2	O3	O4	O5	O6	O7
Zhejiang province							
I	0.5373	0.5506	0.5488	0.3062	0.4795	0.4794	0.5349
M	0.6015	0.4679	0.5069	0.3982	0.5999	0.5921	0.5687
S	0.5222	0.5341	0.4939	0.3622	0.4741	0.4867	0.5492
A	0.5432	0.5432	0.6178	0.3245	0.4607	0.4604	0.5727
Shanghai municipality							
I	0.6975	0.4635	0.6997	0.6267	0.7235	0.7018	0.6618
M	0.5124	0.3879	0.4889	0.4499	0.5133	0.5162	0.4922
S	0.7522	0.4243	0.6740	0.5665	0.7518	0.7561	0.7241
A	0.6006	0.6006	0.5558	0.4202	0.5651	0.5990	0.6351
Jiangsu province						
I	0.5808	0.4093	0.4937	0.3492	0.5020	0.4743	0.5129
M	0.5360	0.3672	0.4754	0.3745	0.4756	0.4265	0.4757
S	0.4999	0.4068	0.4742	0.3192	0.4737	0.4181	0.4799
A	0.5201	0.5201	0.4950	0.3007	0.4070	0.6059	0.4128

## References

[1] CCIEE, Columbia University, Ali Research Institute (2019). Blue Book of Sustainable Development: Evaluation Report on the Sustainable Developement of China.

[2] Kellermann M. (2019). Comprehensive Diagnostic Tool: Annex to the QI Toolkit. International Bank for Reconstruction and Development/The World Bank and Physikalisch-Technische Bundesanstalt (PTB).

[3] Kellermann M. (2019). Ensuring Quality to Gain Access to Global Markets: A Reform Toolkit. International Bank for Reconstruction and Development/The World Bank and Physikalisch-Technische Bundesanstalt (PTB).

[4] Wang J., Wang S., Li S., Feng K. (2019). Coupling analysis of urbanization and energy-environment efficiency: Evidence from Guangdong province. Appl. Energy.

[5] Cui X., Fang C., Liu H., Liu X. (2019). Assessing sustainability of urbanization by a coordinated development index for an Urbanization-Resources-Environment complex system: A case study of Jing-Jin-Ji region, China. Ecol. Indic..

[6] Fan Y., Fang C., Zhang Q. (2019). Coupling coordinated development between social economy and ecological environment in chinese provincial capital cities-assessment and policy implications. J. Clean. Prod..

[7] Li W., Yi P., Zhang D., Zhou Y. (2020). Assessment of coordinated development between social economy and ecological environment: Case study of resource-based cities in Northeastern China. Sustain. Cities Soc..

[8] Salahuddin M., Habib M.A., Al-Mulali U., Ozturk I., Marshall M., Ali M.I. (2020). Renewable energy and environmental quality: A second-generation panel evidence from the Sub Saharan Africa (SSA) Countries. Environ. Res..

[9] Sijm D., Bruijn J.D., Crommentuijn T., Leeuwen K.V. (2001). Environmental quality standards: Endpoints or triggers for a tiered ecological effect assessment approach?. Environ. Toxicol. Chem..

[10] Wang T., Zhou Y., Bi C., Lu Y., Giesy J.P. (2017). Determination of water environment standards based on water quality criteria in China: Limitations and feasibilities. J. Environ. Sci..

[11] Goulden S., Negev M., Reicher S., Berman T. (2019). Implications of standards in setting environmental policy. Environ. Sci. Policy.

[12] Kang N., Kim K.J., Kim J.S., Lee J.H. (2015). Roles of chemical metrology in electronics industry and associated environment in Korea: A tutorial. Talanta.

[13] Orcos R., Pérez-Aradros B., Blind K. (2018). Why does the diffusion of environmental management standards differ across countries? The role of formal and informal institutions in the adoption of ISO 14001. J. World Bus..

[14] Zhang J.H., Chen M. (2015). Assessing the impact of China’s vehicle emission standards on diesel engine remanufacturing. J. Clean. Prod..

[15] Burkhardt J. (2019). The impact of the Renewable Fuel Standard on US oil refineries. Energy Policy.

[16] Leirião L.F.L., Miraglia S.G.E.K. (2019). Environmental and health impacts due to the violation of brazilian emissions control program standards in sao paulo metropolitan area. Transp. Res. Part D Transp. Environ..

[17] Semerjian H.G., Watters R.L. (2000). Impact of measurement and standards infrastucture on the national economy and international trade. Measurement.

[18] Rosin C., Campbell H., Reid J. (2017). Metrology and sustainability: Using sustainability audits in New Zealand to elaborate the complex politics of measuring. J. Rural Stud..

[19] Wang B.Z., Wang H., Guo B.H. (2014). Coupling improved model and the empirical study of regional innovation system. Chin. J. Manag. Sci..

[20] Holzer K., Cottier T. (2015). Addressing climate change under preferential trade agreements: Towards alignment of carbon standards under the Transatlantic Trade and Investment Partnership. Glob. Environ. Chang..

[21] Zhang Y., Shen J., Ding F., Li Y., He L. (2016). Vulnerability assessment of atmospheric environment driven by human impacts. Sci. Total Environ..

[22] He L., Shen J., Zhang Y. (2018). Ecological vulnerability assessment for ecological conservation and environmental management. J. Environ. Manag..

[23] Fan W., Wang H., Liu Y., Liu H. (2020). Spatio-temporal variation of coupling relationship between urbanization development and air quality: A case study of shandong province. J. Clean. Prod..

[24] Hauke J., Kossowski T. (2011). Comparison of values of pearson’s and spearman’s correlation coefficients on the same sets of data. Quaest. Geogr..

[25] Horel J.D. (1981). A Rotated Principal Component Analysis of the Interannual Variability of the Northern Hemisphere 500 mb Height Field. Mon. Wea. Rev..

[26] Denzin N.K. (2012). Triangulation 2.0. J. Mix. Methods Res..

[27] APEC (2017). Guide to Support Quality Infrastructure Incorporation into MSMEs.

